# Protective effect of astaxanthin on indomethacin-induced gastric ulcerations in mice

**DOI:** 10.1007/s00210-024-03206-4

**Published:** 2024-06-28

**Authors:** Mohamed H. Aly, Aya K. Said, Aya M. Farghaly, Dalia A. Eldaly, Dina S. Ahmed, Maram H. Gomaa, Nazih H. Elgebaly, Omar Sameh, Salma K. Elahwany, Tasneem T. Ebrahem, Youssif Sameh, Maha E. Wally

**Affiliations:** 1 Pharmacology Department, Faculty of Pharmacy, The British University in Egypt, Cairo, 11837 Egypt; 2 Health Research Center of Excellence; Drug Research and Development Group, Faculty of Pharmacy, The British University in Egypt, Cairo, 11837 Egypt; 3 Faculty of Pharmacy, The British University in Egypt, Cairo, 11837 Egypt

**Keywords:** Gastric ulcer, Astaxanthin, Inflammation, Indomethacin, Omeprazole, Oxidation

## Abstract

Gastric ulcer disease remains one of the common medical burdens affecting millions worldwide due to its prevalent risk factors with the chronic usage of non-steroidal anti-inflammatory drugs at the top, reportedly through the stimulation of oxidative stress and triggering of inflammatory and apoptotic cascades in the gastric mucosa. Astaxanthin, a dietary keto-carotenoid derived from marine organisms is gaining a wide interest as a nutraceutical for its pronounced antioxidant properties. Here, we aim to examine the potential modulatory role of astaxanthin on indomethacin-induced gastric ulceration in experimental mice. Twenty-four Swiss albino mice were randomly distributed into four groups: a control group, an indomethacin group, and two groups pre-treated with either omeprazole or astaxanthin. The gastric tissues were assessed using gross morphology, ulcer scoring, gastric juice acidity, as well as reduced glutathione (GSH) and malondialdehyde (MDA) levels. Histopathological examination and immunostaining for nuclear factor-kappa B (NF-κB) and caspase-3 levels were also employed. Indomethacin group tended to show a higher number of mucosal ulcerations relative to control and pre-treated groups. The indomethacin group also showed significantly lower GSH levels and higher MDA levels relative to control. Immunostaining of gastric tissue sections showed a higher reactivity to NF-﻿κB and caspase-3 in indomethacin group. Astaxanthin pre-treatment significantly elevated gastric juice pH, normalized GSH levels, and lowered the indomethacin-induced elevations in MDA, NF-﻿κB, and caspase-3 levels. These results indicate that astaxanthin exhibits a comparable protective effect to omeprazole, against indomethacin-induced gastric ulceration. This anti-ulcerogenic effect could be mediated through its antioxidant, anti-inflammatory, and anti-apoptotic modulatory activities.

**Graphical Abstract:**

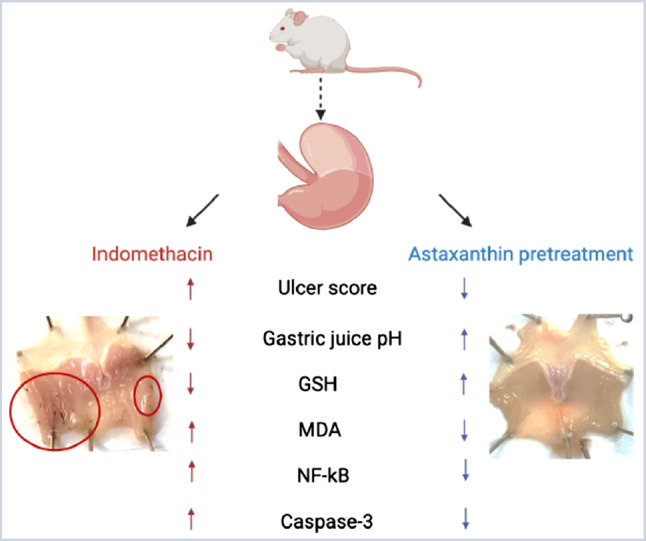

## Introduction

Gastric ulcer disease (GUD) is a common digestive tract disorder affecting around four million people per year worldwide (Abbasi-Kangevari et al. 2022) with the global prevalence increasing 25.82% between 1990 and 2019 (Lanas and Chan 2017). Despite the eradication of the main risk factor associated with gastric ulcer over the past decades namely *Helicobacter pylori* infection, this upsurge in the recorded cases has been attributed to an increased prevalence of other risk factors such as the chronic usage of non-steroidal anti-inflammatory drugs (NSAIDs), selective serotonin re-uptake inhibitors (SSRIs), and gastric bypass operations (Xie et al. 2022).

NSAIDs accounted for around 10% of the most prescribed medications in the early 2000s (Wongrakpanich et al. 2018). Due to their profound analgesic, antipyretic, and anti-inflammatory properties, they are commonly used for pain management and inflammation associated with rheumatic disorders, dysmenorrhea (Marjoribanks et al. 2010), gout (van Durme et al. 2021), and migraines (Pardutz and Schoenen 2010). However, their wide clinical usage is hindered by their pronounced side effects, specifically gastric ulcerations, renal damage, and cardiovascular thrombotic events (Drini 2017; Bindu et al. 2020).

A wide array of pharmacological therapies exists in the market for the treatment of GUD (Kuna et al. 2019). Proton pump inhibitors (PPIs) act through blocking the H+/K+ ATPases in the parietal cells of the stomach lining (Strand et al. 2017; Shanika et al. 2023). Histamine receptor-2 antagonists (H2Ras) antagonize the histamine-2 receptor and thus block the acid-secretory effect of histamine (Fox and Muniraj 2016). Mucosal protectants provide mucosal protective barriers, and antacids reduce gastric acidity (Haruma and Ito 2003). Side effects of these medications range from hypersensitivity reactions, increased risk of enteric infections, decrease of vitamin absorption up to dementia, and an elevated risk for gastric cancer (Ahmed 2020; Yibirin et al. 2021).These prominent side effects combined with the high relapse rates (Seo et al. 2016; Alsinnari et al. 2022) prompt the need to screen for natural products with a prophylactic potential against GUD.

Astaxanthin is a keto-carotenoid belonging to the xanthophylls class, naturally produced by the microalgae *Haematococcus pluvialis* and found abundantly in the algae-feeding aquatic red-orange organisms such as shrimps, crabs, and salmon (Nishida et al. 2022). It is known for its profound antioxidant properties owing to its unique chemical structure (Davinelli et al. 2022) with its high free-radical quenching ability. This allowed astaxanthin to demonstrate multiple antioxidant protective properties namely neuroprotective, cardioprotective, muscle-protective, and anti-aging properties (Fakhri et al. 2018). Astaxanthin has been shown to increase the antioxidant body defenses by enhancing the expression of superoxide dismutase (SOD), glutathione peroxidase (GPx), and catalase (CAT) (Guerin et al. 2003). Additionally, it has revealed an anti-inflammatory potential by decreasing the expression of tumor necrosis factor (TNF)-alpha, Interleukin (IL)-1B, and nuclear factor kappa B (NF-κB) (Ohgami et al. 2003; Suzuki et al. 2006; Speranza et al. 2012). Although the direct contribution of astaxanthin’s anti-inflammatory effects to GUD prevention remains elusive, the aforementioned properties hint at a possible protective role of astaxanthin on NSAID-induced GUD, specifically that of indomethacin which is characterized by extensive oxidative damage and pronounced inflammation of gastric mucosa (Suleyman et al. 2010).

Hence, this study aims to explore the potential protective effect of astaxanthin on indomethacin-induced gastric ulcerations in experimental mice and shed light on its prospective underlying mechanism.

## Materials and methods

### Drugs and chemicals

Astaxanthin was purchased from Otsuka Pharmaceuticals Co., Ltd (Japan); indomethacin was purchased from Nile Company for Pharmaceutical and Chemical Industries (Cairo, Egypt) under license of Chiesi Farmaceutici S.P.A. Pharma (Italy). Omeprazole was purchased from Global NAPI Pharmaceuticals (Cairo, Egypt). Tween 80 was purchased from Sigma Chemical Co. (St. Louis, MO, USA). All other chemicals and reagents used in this study were of the highest purity grade.

### Animals

Male Swiss albino mice, average weight 25 to 35 g, were acquired from the animal house of the British University in Egypt (BUE) and maintained on a 12-h light/dark cycle at temperatures of (22 ± 3 °C) and 60% humidity with free access to water and food. Animals were allowed to acclimatize for a period of 1 week before the start of the experiment. All the animal experiments and care protocols were approved by the Research Ethics Committee of the Faculty of Pharmacy at the British University in Egypt (BUE) reference no “EX-2308.”

### Experimental design

**Fig. 1:**
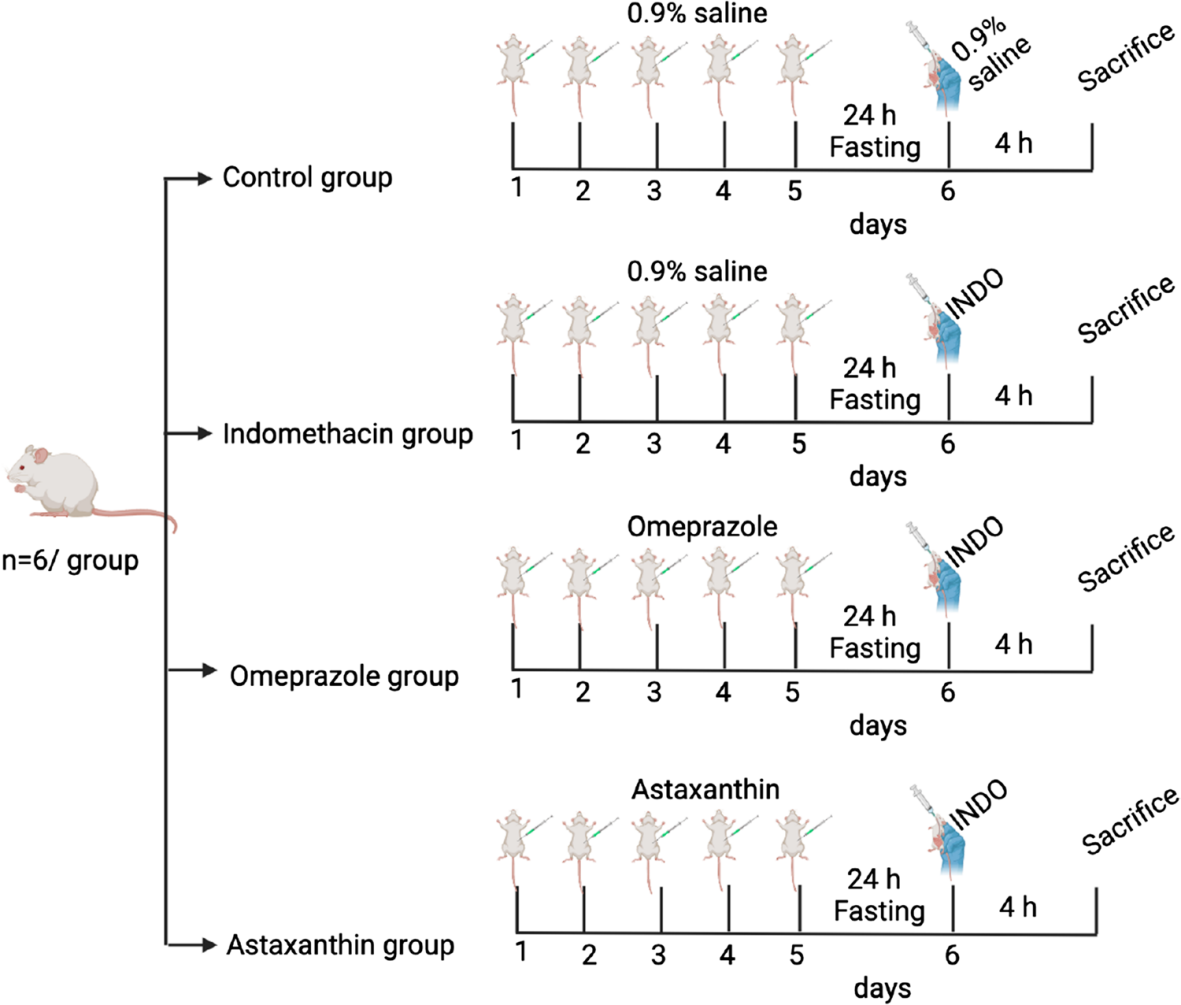
Experimental design. Experimental design includes four animal groups: (A) control group which received only the drug vehicles (0.9% saline), (B) indomethacin group which received a single oral dose (SOD) of indomethacin (40 mg/kg) for induction of gastric ulcer, (C) omeprazole group which received I.P. omeprazole injections (20 mg/kg) as standard prophylactic agent for 5 consecutive days followed by SOD of indomethacin (40 mg/kg), (D) astaxanthin group which received I.P. astaxanthin injections (40 mg/kg) as a potential prophylactic agent for 5 consecutive days followed by SOD of indomethacin (40 mg/kg). All groups were sacrificed 4 h after indomethacin injection for ulcer scoring and other analysis. This figure was created with BioRender.com

Mice were randomly allocated using the GraphPad online number generator into four groups (*n* = 6 per group) as follows (Fig. 1): Group 1 (control group) received IP injections of 0.9% saline (drug vehicle) for 5 consecutive days then subjected to 24 h fasting (Ma et al. 2022) before receiving an oral solution of 0.9% saline (indomethacin vehicle). Group 2 (indomethacin group) received IP injections of 0.9% saline (drug vehicle) for 5 consecutive days then subjected to 24 h fasting before receiving a single oral dose (SOD) of indomethacin (40 mg/kg) (Ma et al. 2022) dissolved in 0.9% saline. Group 3 (omeprazole group) received IP injections of omeprazole (20 mg/kg)(Ma et al. 2022) for 5 consecutive days then subjected to 24 h fasting before receiving a single oral dose (SOD) of indomethacin (40 mg/kg) dissolved in 0.9% saline. Group 4 (astaxanthin group) received IP injections of astaxanthin (40 mg/kg) (Zhang et al. 2018) for 5 consecutive days then subjected to 24 h fasting before receiving a single oral dose (SOD) of indomethacin (40 mg/kg) dissolved in 0.9% saline. For each daily treatment, the order of subjects receiving treatment within each group was randomly shuffled using the GraphPad online number generator.

Four hours after indomethacin administration, mice were subjected to CO_2_ euthanasia followed by decapitation. After sacrificing the mice, an incision was made in the skin and underlying muscles; the stomach was located, and a clamp was placed around the pyloric end. A small incision was made on the esophageal end, and the gastric juice was drained and collected in a centrifugation tube for measurement of gastric juice acidity (El-Ashmawy et al. 2016). The stomachs were cut along the greater curvature, washed with cold saline, and examined morphologically for ulcer scoring (Belayneh et al. 2021). Each stomach was divided into two identical halves; one half was stored in 10% formalin for histopathological examination, and the other half was snap-freezed using liquid nitrogen then stored at −80 °C for biochemical measurements (Ma et al. 2022). Dead animal bodies were kept in biohazard bags in −20 °C freezer and finally incinerated by specialized waste disposal companies.

### Determination of gastric juice acidity

The gastric juice from each mouse was collected and diluted with 5 ml distilled water and centrifuged (Thermo scientific, Germany) at −4 °C for 10 min at 1000 rpm (El-Ashmawy et al. 2016), and the pH was measured using a pH meter (Jenway, UK). Gastric juice extraction was successful for 16 out of 24 mice.

### Morphological examination and determination of ulcer score

For morphological gross assessment, each stomach was washed with cold saline and placed on a cork plate with pins and photographed with a digital camera (AbdelAziz et al. 2021). An ulcer score was given to each mouse depending on the severity of ulcerations using the following scoring system: normal stomach color (0), hyperemia (0.5), spot ulcers (1), hemorrhagic streak (1.5), deep ulcers (2), and perforation (3) (Belayneh et al. 2021).

### Histopathological examination and immunohistochemical staining

Gastric tissues were fixed in 10% formalin then submerged in serial ethanol dilutions and cleared with xylene before being embedded in paraffin blocks. Tissue blocks were sliced into 4-µm-thick sections. For histological examinations, sections were stained with hematoxylin and eosin, mounted using DPX mountant (Sigma-Aldrich, USA, Cat. No. 06522) and finally covered. Alternatively, for the immunohistochemical staining, sections were rehydrated, mixed with boiling citrate buffer, incubated with peroxidase blocking serum for 10 min, and rinsed with phosphate buffered saline (PBS) before another 10-min incubation period with a protein blocking serum. Subsequently, primary antibodies against NF-κB (Cell signaling Technology, USA, Cat. No.: 8242, Dilution 1:500) or caspase-3 (Abclonal, USA, Cat. No.: A2156, Dilution 1:100) were added for 30 min at room temperature followed by a 20-min incubation with a secondary HRP-labelled antirabbit polymer (Agilent Technologies, USA, Cat. No. K4003) for 20 min before the final addition of 3,3ʹ-diaminobenzidine (DAB) (Sigma-Aldrich, USA, Cat. No. D12384) (AbdelAziz et al. 2021). Finally, counterstaining with Harris’s hematoxylin was done before the sections were dehydrated using serial ethanol dilutions, cleared with xylene, DPX mounted, and covered. A combined microscopic imaging system (Olympus Corporation, Japan, Cat No. CX 41RF and SC100) was used for image acquisition.

### Determination of oxidative stress markers

Frozen stomach tissues were homogenized on ice in commercially available buffers using a tissue homogenizer (Sonics, USA), and homogenates were evaluated for the levels of reduced glutathione (GSH) and lipid peroxides/malondialdehyde (MDA) using colorimetric kits from Biodiagnostics Co. (Cairo, Egypt) as per the manufacturer’s instructions.

#### Determination of gastric GSH levels

Reduced glutathione (GSH) in the gastric tissues was determined based on the reduction of 5,5ʹ-dithiobis(2-nitrobenzoic acid) (DTNB) with GSH to produce a yellow compound where the reduced chromogen is directly proportional to the GSH concentration in the sample (Beutler et al. 1963).Gastric tissue (0.1g) was homogenized in 1 ml ice-cold (50 mM potassium phosphate with 1 mM EDTA, PH 7.5) using a tissue homogenizer. Then, the samples were centrifugated at 4000 rpm for 15 min at 4 °C. Trichloroacetic acid (TCA, 1 ml) was added to 0.5 ml of the supernatant and then centrifugated at 3000 rpm for 15 min at room temperature. DTNB reagent (0.1 ml) was added to 0.5 ml of the supernatant, and the absorbance was measured at 405 nm using a spectrophotometer (Jenway, Fischer Scientific, Cat No. 83056-02) against blank after 5–10 min.

#### Determination of gastric MDA levels

Malondialdehyde (MDA) in the gastric tissues was determined based on the reaction of MDA with thiobarbituric acid (TBA) in acidic medium at 95 °C for 30 min to form a reactive pink product whose absorbance in measured at 534 nm (Ciuti and Liguri 2017) . Gastric tissue (0.1g) was homogenized in 1 ml ice-cold (50 mM potassium phosphate, PH 7.5) using a tissue homogenizer. Then, the samples were centrifugated at 4000 rpm for 15 min at 4 °C. TBA (1 ml) was added to 0.5 ml of the supernatant and heated in boiling water for 30 min. The samples were allowed to cool; then, the absorbance was measured against blank at 534 nm using a spectrophotometer (Jenway, Fischer Scientific, Cat No. 83056-02).

#### Statistical analysis

The resulting data were plotted as mean value ± standard deviation (SD). Ulcer score was evaluated as median ± individual values using Kruskal–Wallis test. Multiple group analysis was statistically evaluated by one-way analysis of variance (ANOVA) followed by post hoc Tukey–Kramer or Newman–Keuls tests. Statistical significance was deemed at *P* values ≤ 0.05. No exclusions were made. All graphical representations and statistical analyses were performed using GraphPad Prism software, version 5.00 (GraphPad Software, Inc. La Jolla, CA, USA). Groups were assigned numbers, and the treatments were assigned letters by M.E.W; other investigators were blinded to the identity of each during allocation, treatment, sampling, and analysis.

## Results

### Effect of astaxanthin on gastric morphology and ulcer score

**Fig. 2:**
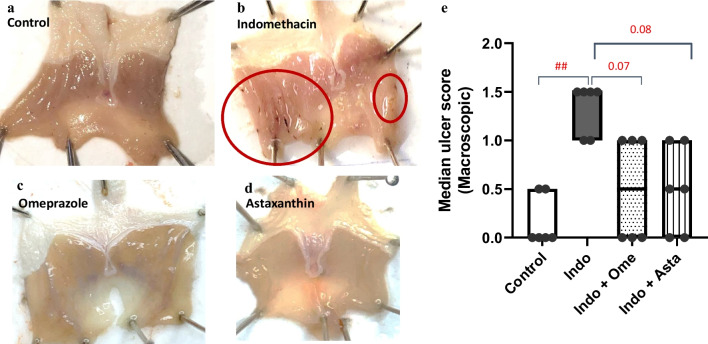
Effect of astaxanthin on gastric tissue gross morphology and ulcer score. **a**–**d** Representative images of dissected gastric tissue for each group: **a** control group, **b** indomethacin group with red circles showing macroscopically detected ulcers, **c** omeprazole pre-treated group, and **d** astaxanthin pre-treated group with no visible gastric ulceration. **e** Ulcer scoring for the dissected gastric tissue for each group. *P* values were determined using Kruskal–Wallis test with Dunn’s correction. Data presented as median ± individual values. (*n* = 6) ^##^*P* < 0.01

Control group showed normal gastric mucosa with a median of zero estimated using the ulcer scoring system (Fig. 2a and e). Pre-treatment with astaxanthin or omeprazole showed no visible gastric ulcerations (Fig. 2c and d) and tended to decrease the ulcer score (Fig. 2e) compared to indomethacin group, which showed a significantly higher number of mucosal ulcerations and blood streaks relative to control (Fig. 2b).

### Effect of astaxanthin on gastric juice pH

**Fig. 3:**
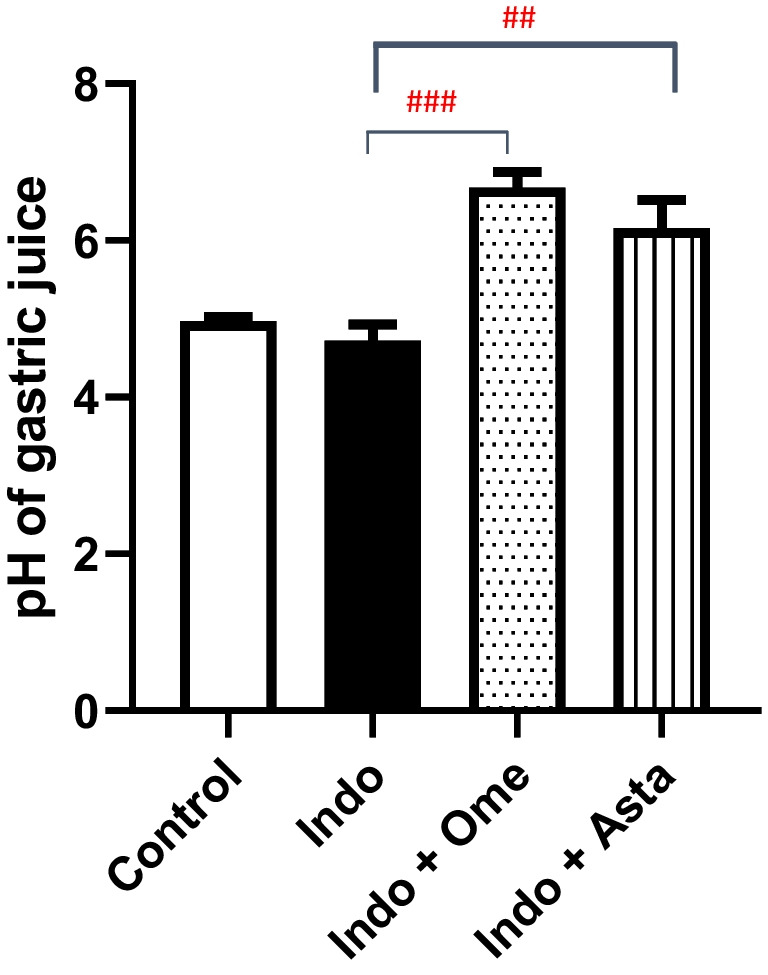
Effect of astaxanthin on gastric juice acidity. pH of gastric juice extracted from each group. *P* values were determined using one-way ANOVA followed by Tukey’s multiple comparisons. Data are presented as mean ± SD (*n* = 4). ^##^*P* < 0.01; ^###^*P* < 0.001

Control group showed mean gastric juice pH of 4.97, whereas indomethacin group showed a 5% decrease with a relatively lower gastric juice pH of 4.72. Moreover, pre-treatment with astaxanthin or omeprazole significantly elevated the gastric juice pH by 1.3 and 1.4 folds to 6.15 and 6.67, respectively (Fig. 3).

### Effect of astaxanthin on histological features of gastric tissue

**Fig. 4:**
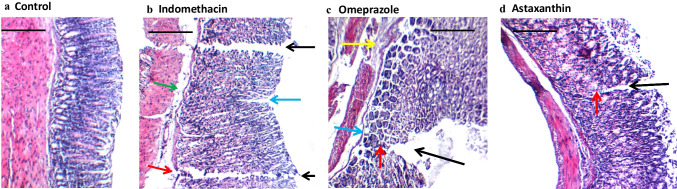
Effect of astaxanthin on microscopic gastric mucosal damage. **a**–**d** Histopathological examination of gastric tissue extracted from each group using hematoxylin and eosin (H and E) stain. **a** Control: gastric wall showing average mucosal thickness, submucosa, and musculosa with the preservation of superficial layer. **b** Indomethacin: gastric wall showing two deep ulcers (black arrow) destructing muscularis mucosa (red arrow), another superficial ulcer (blue arrow), and mildly edematous submucosa (green arrow). **c** Omeprazole: Gastric wall shows superficial ulcer (black arrow) involving more than half of mucosal thickness (red arrow), intact muscularis mucosa (blue arrow), and mildly edematous submucosa (yellow arrow). **d** Astaxanthin: gastric wall showing superficial ulcer (black arrow) involving more than half of mucosal thickness (red arrow) with average submucosa, and musculosa. High power resolution ×200; scale bar= 40 µm

Histopathological examination using hematoxylin and eosin (H and E) stain revealed normal intact gastric wall in the control group (Fig. 4a; Table 1), whereas indomethacin group displayed both deep and superficial ulcerations in the gastric wall (Fig. 4b; Table 1). Pre-treatment with astaxanthin or omeprazole revealed the presence of only superficial ulcers with an intact muscularis mucosa (Fig. 4c and d; Table 1).

**Table 1:** Histopathological analysis of gastric tissue in different treatment groups

	Control	Indomethacin	Omeprazole	Astaxanthin
Erosion/ulceration	-	++	-	-
Inflammatory cell infiltrates	-	++	+	+
Congested blood Vessel	-	+	-	-
Edema	-	+	+	-

*-* nil, *+* mild (less than 15% of examined samples), *++* moderate (16–35% of examined samples), *+++* severe (more than 35% of examined examples)

### Effect of astaxanthin on gastric GSH levels

**Fig. 5:**
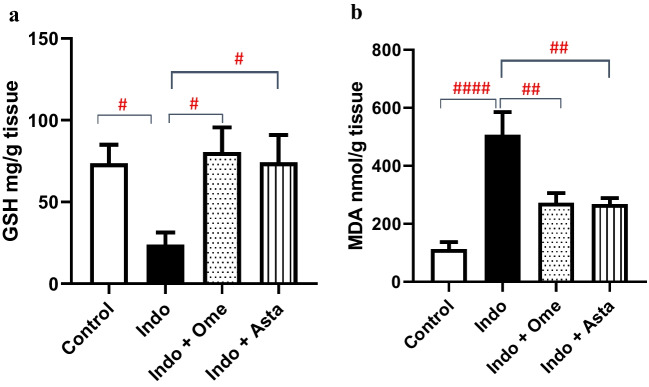
Effect of astaxanthin on indomethacin induced oxidative stress. **a** Reduced glutathione (GSH) levels in gastric tissue extracted from each group. **b** Lipid peroxidation by-product malondialdehyde (MDA) levels in gastric tissue extracted from each group. *P* values were determined using one-way ANOVA followed by Newman–Keuls multiple comparisons. Data are presented as Mean ± SD (*n* = 6). ^#^*P* < 0.05; ^##^*P* < 0.01; ^####^*P* < 0.0001

Low reduced glutathione (GSH) levels serve as an indication of high oxidative stress levels within the gastric tissue (Bhattacharyya et al. 2014; Pérez et al. 2017). In line with this, the indomethacin group significantly lowered the levels of GSH by 67.5% relative to the control group, whereas pre-treatment with astaxanthin or omeprazole significantly elevated GSH levels by 3.1 and 3.4 folds, respectively, compared to indomethacin group (Fig. 5a).

### Effect of astaxanthin on gastric MDA levels

High malondialdehyde (MDA) levels reflect excessive lipid peroxidation in gastric tissue (Kwiecien et al. 2014). In line with this, the indomethacin group significantly increased the levels of MDA by 4.5 folds relative to the control group, whereas pre-treatment with astaxanthin or Omeprazole significantly lowered the MDA levels by 47% and 46%, respectively, compared to indomethacin group (Fig. 5b).

### Effect of astaxanthin on NF-κB gastric expression

**Fig. 6:**
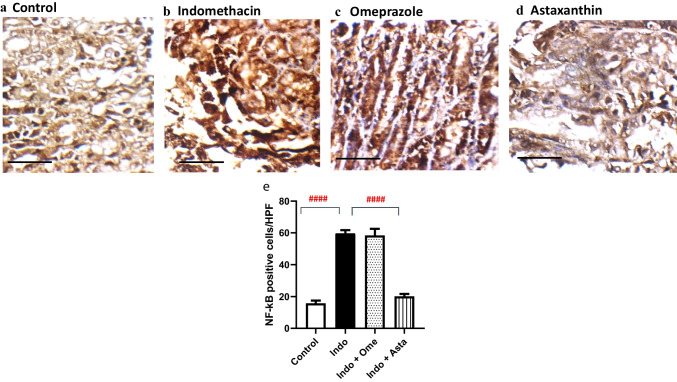
Effect of astaxanthin on NF-﻿κB immunohistochemical expression. Representative photomicrographs of gastric wall sections for each group. **a** Control: gastric wall shows baseline reactivity to NF-﻿κB antibody in superficial layers of mucosa. **b** Indomethacin: superficial mucosa shows relatively more dense expression levels of NF-﻿κB. **c** Omeprazole and **d** astaxanthin: NF-﻿κB expression tends to be relatively less dense expression in the superficial mucosa than that of indomethacin group. High power resolution ×400; scale bar = 20 µm. **e** Number of immune-positive cells per high power field; *P* values were determined using One-way ANOVA followed by Tukey’s multiple comparisons. Data are presented as Mean ± SD (*n* = 3). ^####^*P* < 0.0001

High nuclear factor-kappa B (NF-κB) levels indicate an existing inflammatory cascade within the gastric tissue (Takahashi et al. 2001; Sokolova and Naumann 2017). Similarly, indomethacin group displayed a significantly higher expression of NF-κB in the superficial mucosa of the gastric wall by 3.8 folds relative to the control group (Fig. 6a, b, and e)., whereas pre-treatment with astaxanthin showed a significantly lower expression profile by 66.5% compared to that of indomethacin group (Fig. 6d and e) and by 65.7% relative to that of the omeprazole group (Fig. 6c and e).

### Effect of astaxanthin on caspase-3 gastric expression

**Fig. 7:**
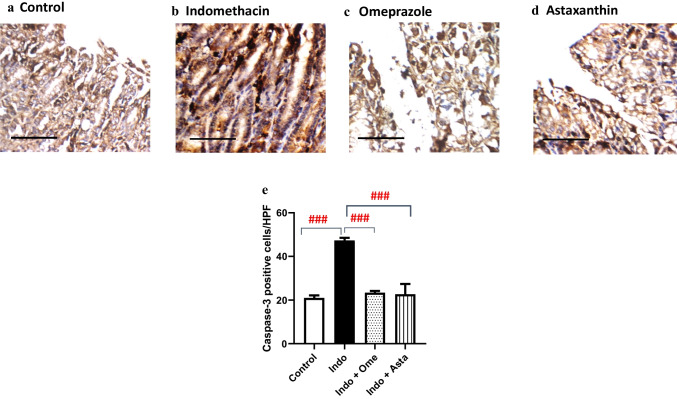
Effect of astaxanthin on caspase-3 immunohistochemical expression. Representative photomicrographs of gastric wall sections for each group. **a** Control: superficial layers of mucosa in the gastric wall shows baseline reactivity to caspase-3 immunostaining. **b** Indomethacin: superficial mucosa shows relatively more dense expression levels of caspase-3. **c** Omeprazole and **d** astaxanthin: caspase-3 expression tends to show relatively less dense expression in the superficial mucosa than that of indomethacin group. High power resolution ×400; scale bar = 40 µm. **e** Number of immune-positive cells per high power field; *P* values were determined using one-way ANOVA followed by Tukey’s multiple comparisons. Data are presented as mean ± SD (*n* = 3). ^###^*P* < 0.001

Indomethacin contributes to high levels of the pro-apoptotic caspase-3 in the gastric tissue (Gebril et al. 2020), which serves as a key indicator to the induction of the apoptotic cycle in the gastric tissue. In line with this, indomethacin group showed a significantly elevated expression of caspase-3 in the superficial mucosa of the gastric wall by 2.3 folds relative to the control group (Fig. 7a, b and e), whereas pre-treatment with astaxanthin or omeprazole showed a significantly less dense expression profile than that of indomethacin group by 52.1% and 50.7% respectively (Fig. 7c, d, and e).

## Discussion and conclusion

GUD remains one of the most common disorders affecting 5–10% of the general population despite the availability of a multiple treatment options such as proton pump inhibitors, H_2_ receptor antagonists, antacids, and mucosal barriers (Kuna et al. 2019; Yibirin et al. 2021). Complex mechanisms underlie the pathogenesis of GUD with the most prominent feature being an imbalance between the gastroprotective factors ranging from the mucosal barrier, endogenous antioxidants, bicarbonate secretion to cell regeneration, and the aggressive factors, such as gastric acid secretion, pepsin secretion, and reactive oxygen species (Højgaard et al. 1996; Alves Araujo de Lima et al. 2022). Unresolved GUD could lead to GIT perforations, bleeding, and gastric cancer (Ahmed 2020). The most common risk factors for GUD are infection with *Helicobacter pylori* and the chronic usage of NSAIDs (Xie et al. 2022).

Indomethacin is a potent NSAID used in the treatment of arthritic conditions including rheumatoid arthritis, ankylosing spondylitis, and osteoarthritis; however, its clinical usage is limited by its damaging gastric effects (Suleyman et al. 2010). As with other NSAIDs, indomethacin induces gastric mucosal ulcerations by pharmacologically inhibiting the cyclooxygenase (COX) enzyme, curbing the synthesis of gastro-protective prostaglandins, decreasing the epithelial cell renewal as well as the mucosal blood flow, and increasing the acid back diffusion (Fornai et al. 2011; Drini 2017). Parallel to this, indomethacin has shown prostaglandin-independent effects mediated through an increase in pro-inflammatory mediators, reactive oxygen species, and neutrophil infiltration of the gastric tissue, eventually resulting in apoptosis and gastric injury (Musumba et al. 2009). In line with previous studies (Katary and Salahuddin 2017; Ock et al. 2017; Ko et al. 2020; El-Sisi et al. 2020; Eraslan et al. 2020), we have shown that indomethacin caused severe gastric ulcerations as evident by gross morphology (Fig. 2b), ulcer scoring (Fig. 2e), and histopathological examination (Fig. 4b; Table 1).

Astaxanthin, a now popular nutraceutical, has been approved as a dietary supplement by the US Food and Drug Administration (FDA) for its wide range of health benefits mediated by its powerful antioxidant properties that has been reported to be even stronger than vitamin E and beta carotenes (O’Connor and O’Brien 1998). In addition to the reported antioxidant ability, it has been shown to possess potent anti-inflammatory and anti-apoptotic effects (Fakhri et al. 2018). The current study aimed to unveil whether these effects could also modulate a potential protective role for astaxanthin against indomethacin-induced gastric ulcerations in mice.

Interestingly, pre-treatment with astaxanthin curbed the indomethacin-induced gastric damage as revealed with the improved morphology (Fig. 2d), ulcer score (Fig. 2e), and histopathological features of a less damaged gastric mucosa (Fig. 4d). Astaxanthin’s comparable results to omeprazole pre-treatment suggest a similar protective effect of astaxanthin on the gastric mucosa by decreasing the incidence of deep ulcerations (Fig. 2c, d and 4c, d; Table 1). Furthermore, the significantly higher gastric juice pH observed in both astaxanthin and omeprazole pre-treated groups compared to indomethacin group suggests a potential gastroprotective mechanism of astaxanthin by decreasing the overall gastric juice acidity (Fig. 3).

Owing to its highly polar chemical structure, astaxanthin possessed a broad range of antioxidant effects from neutralizing singlet oxygen molecules to scavenging of free radicals and was found to diminish lipid peroxidation and preserve the integrity of cellular membranes (Fakhri et al. 2018). Among the body’s antioxidant defense systems (Nakajima et al. 2008; Otton et al. 2010; Marin et al. 2011; Yin et al. 2021), astaxanthin has been also shown to increase the levels of protective GSH (Yin et al. 2021), a powerful intracellular guard against the reactive oxygen species that prevent oxidative damage to the cells (Bhattacharyya et al. 2014). By quenching free radicals, astaxanthin provides a protective effect against lipid peroxidation as marked by decreasing the levels of MDA, the end-product of lipid peroxidation (Kwiecien et al. 2014). In line with these reports, both astaxanthin and omeprazole pre-treated groups normalized the indomethacin-induced depletion of GSH stores (Fig. 5a), reflecting their potent antioxidant effects. Furthermore, the spike in MDA levels observed in the indomethacin group has been reduced in the two pre-treated groups (Fig. 5b), highlighting their role against lipid peroxidation and the damaging reactions of an unopposed elevation of oxidative stress.

Astaxanthin exhibits a broad immune-modulatory activity via multiple pathways as seen in various disease models (Guerin et al. 2003; Ohgami et al. 2003; Suzuki et al. 2006; Speranza et al. 2012). Most importantly, it has been shown to inhibit the activation of the transcription factor NF-κB, which controls the expression of various inflammatory products (Lawrence 2009). Consistent with previous data, pre-treatment with astaxanthin significantly normalized the levels of pro-inflammatory NF-κB signaling in the gastric tissue (Fig. 6). This suggests a potential anti-inflammatory effect behind its exhibited gastro-protective potential.

Existing literature demonstrates an indomethacin-induced elevation in pro-apoptotic signaling molecules (Katary and Salahuddin 2017; Ock et al. 2017; Ko et al. 2020; El-Sisi et al. 2020; Eraslan et al. 2020), specifically caspase-3, a key pro-apoptotic executioner. In our study, we reveal that pre-treatment with astaxanthin significantly lowered the levels of caspase-3 in the gastric tissue (Fig. 7), consistent with previous reports (Zhang et al. 2018), which reflects a possible antiapoptotic potential for astaxanthin.

Taken together, our study has demonstrated that indomethacin significantly induced gastric ulceration in mice probably through its depletion of GSH stores, which hinders the antioxidant defenses and induces lipid peroxidation as evidenced by the elevated gastric MDA levels. Furthermore, indomethacin caused an upregulation of inflammatory transcription factor “NF-κB” and apoptosis executioner “caspase-3” in the superficial mucosa of the gastric wall. As opposed to indomethacin, pre-treatment with astaxanthin tended to show anti-ulcerogenic effects probably due to the elevation of gastric juice pH as well as the normalization of GSH, MDA, NF-κB, and caspase-3 gastric tissue levels.

While our study has shed light on a prospective protective function for astaxanthin against GUD, further mechanistic studies are needed to rule out the major downstream key players involved in this effect. Specifically, a comprehensive analysis for the inflammatory and apoptotic mediators could offer an in-depth understanding of the cascade of molecular events targeted by astaxanthin in terms of its protection. Future directions could further include a combination pre-treatment group receiving both astaxanthin and omeprazole to highlight the presence of any synergistic or additive effects, which could lower omeprazole dose and minimize the side effects. Finally, the potential antiulcerogenic effect of astaxanthin should also be confirmed using other models of gastric ulcer.

## Data Availability

No datasets were generated or analysed during the current study.
